# AI-Assisted Assessment of Wound Tissue with Automatic Color and Measurement Calibration on Images Taken with a Smartphone

**DOI:** 10.3390/healthcare11020273

**Published:** 2023-01-16

**Authors:** Sawrawit Chairat, Sitthichok Chaichulee, Tulaya Dissaneewate, Piyanun Wangkulangkul, Laliphat Kongpanichakul

**Affiliations:** 1 Department of Biomedical Sciences and Biomedical Engineering, Faculty of Medicine, Prince of Songkla University, Songkhla 90110, Thailand; 2 Research Center for Medical Data Analytics, Faculty of Medicine, Prince of Songkla University, Songkhla 90110, Thailand; 3 Department of Rehabilitation Medicine, Faculty of Medicine, Prince of Songkla University, Songkhla 90110, Thailand; 4 Division of General Surgery, Department of Surgery, Faculty of Medicine, Prince of Songkla University, Songkhla 90110, Thailand; 5 Division of Plastic and Reconstructive Surgery, Department of Surgery, Faculty of Medicine, Prince of Songkla University, Songkhla 90110, Thailand

**Keywords:** chronic wound, wound care, image segmentation, deep learning, color calibration

## Abstract

Wound assessment is essential for evaluating wound healing. One cornerstone of wound care practice is the use of clinical guidelines that mandate regular documentation, including wound size and wound tissue composition, to determine the rate of wound healing. The traditional method requires wound care professionals to manually measure the wound area and tissue composition, which is time-consuming, costly, and difficult to reproduce. In this work, we propose an approach for automatic wound assessment that incorporates automatic color and measurement calibration and artificial intelligence algorithms. Our approach enables the comparison of images taken at different times, even if they were taken under different lighting conditions, distances, lenses, and camera sensors. We designed a calibration chart and developed automatic algorithms for color and measurement calibration. The wound area and wound composition on the images were annotated by three physicians with more than ten years of experience. Deep learning models were then developed to mimic what the physicians did on the images. We examined two network variants, U-Net with EfficientNet and U-Net with MobileNetV2, on wound images with a size of 1024 × 1024 pixels. Our best-performing algorithm achieved a mean intersection over union (IoU) of 0.6964, 0.3957, 0.6421, and 0.1552 for segmenting a wound area, epithelialization area, granulation tissue, and necrotic tissue, respectively. Our approach was able to accurately segment the wound area and granulation tissue but was inconsistent with respect to the epithelialization area and necrotic tissue. The calibration chart, which helps calibrate colors and scales, improved the performance of the algorithm. The approach could provide a thorough assessment of the wound, which could help clinicians tailor treatment to the patient’s condition.

## 1. Introduction

Chronic wounds are a major public health problem worldwide that affect people’s health and daily activities, incur costs, and potentially lead to mortality. More than 6.5 million people have chronic wounds, which are estimated to cost more than 25 billion dollars per year in the United States. The prevalence of chronic wounds was 2.21 per 1000 population [1]. Healthcare costs for chronic wounds were estimated to be around GBP 2.3 billion to GBP 3.1 billion per year in the United Kingdom alone [2].

When a wound occurs, the skin of the body breaks down and loses its barrier function, posing a high risk of infection to the injured area [3]. Wounds can be divided into acute and chronic wounds depending on how long they take to heal. Acute wounds progress through the normal stages of wound healing and exhibit definitive signs of healing within four weeks. If wound healing does not make much progress within four weeks, it is referred as a chronic wound or non-healing wound. Many factors can interrupt the normal wound healing process. These wounds often occur in people with comorbidities such as diabetes and obesity. The treatment of these wounds is extremely expensive. Patients with a chronic wound require more extensive wound care than acute wounds [4]. They need to see a physician regularly to follow the progress of wound healing. Wound management must follow best practices to promote healing and minimize wound complications.

Clinical guidelines are one of the most important components of good wound care. Current wound care guidelines emphasize the need for regular documentation, including wound size, as well as the need to determine a percentage or rate of healing. The guidelines rely on ongoing wound measurements to determine whether a change in treatment is needed if wounds are not healing as expected. If healing does not occur, alternative therapies may be needed. To ensure evidence-based best practice, accurate and reliable wound measurement must be a regular part of wound care to optimize patient care and outcomes.

Wound measurement tools play an important role in helping physicians measure, monitor, and develop a wound treatment strategy. The tools can be used to evaluate wound margin and wound tissue area (e.g., granulation tissue, necrotic tissue, and epithelialization area), which can serve as indicators of wound healing [4]. The traditional method is a ruler method. The wound is usually measured first in its length, then in its width, and in its depth by inserting a cotton swab into the deepest part of the wound. The ruler method is simple and straightforward, but time-consuming and often inaccurate, as it is difficult to take measurements and estimate the wound area. It also carries a risk of infection to the patient during the measurement [5].

In wound care practice, there are several assessment tools commonly used by healthcare professionals. The Braden, Norton, and Waterlow scales are used to determine the risk of developing a pressure ulcer. The Wagner system is used to classify different grades of diabetic foot ulcer. The resulting scores can help physicians plan treatment and improve patient outcomes, including faster healing, less pain and discomfort, and a lower risk of infection. However, these scales are usually subjective, and the score obtained depends on the professional performing the evaluation [6].

The digital measurement of wound images with devices or applications has recently been used to measure the wound area [7]. This method solves the problems of the traditional method because it does not come into direct contact with the wound [8]. Currently, there are only a few specially developed devices on the market. Some of them only allow the measurement of the wound area, while others allow the measurement of different tissue types [7,9]. Some devices also require the physician to manually set seeding points for each wound tissue type for accurate measurements.

Several recent studies have used artificial intelligence (AI) for wound assessment to overcome the problem of the ruler method. AI has the potential to develop a fully automated wound measurement tool that can provide accurate measurements of different wound features that can be used for wound assessment and treatment planning [8,10,11]. However, the development of an AI-assisted wound assessment tool requires a dataset with good quality and a large number of data, but only wound datasets with a small number of images are currently publicly available. Most datasets only contain the labeling of the wound area, while other features that can be used for wound assessment, such as the labeling of granulation tissue, necrotic tissue, and epithelialization area, are missing. These three different tissue types in the wound bed play an important role in assessing and evaluating the wound healing progression and predicting the wound healing outcome [8]. In addition to approaches that use AI and large datasets to train algorithms, there are also methods based on superpixel segmentation [12], region growing [13], and expert systems [14] that do not require training. These approaches rely on the knowledge and expertise of the individuals that use the systems.

This study aimed to develop an approach for wound assessment based on deep learning. Our algorithms are able to segment the wound area, granulation tissue, necrotic tissue, and epithelialization area from wound images captured with a mobile phone. The algorithms could be further developed into a mobile application that provides physicians with a practical tool for wound assessment.

Our contributions are as follows. First, we purposefully collected a dataset of wound images for this study. All wound images were labeled by three physicians who provided tracings for wound area, granulation tissue, necrotic tissue, and epithelialization area. Second, we employed a color and measurement calibration chart and developed an automatic calibration procedure to tackle the negative effects of color variation in images taken in unconstrained conditions. Third, we compared the tracings among physicians and between physicians and the algorithm and provided an in-depth analysis of the results.

Our paper is organized as follows. Section 2 provides background information and a review of the relevant literature. Section 3 describes the study design, data collection methods, and procedures used in the study. Section 4 presents our findings. Section 5 discusses the implications and limitations of our findings. Section 6 concludes the paper.

## 2. Literature Review

Wound is defined as any injury to the loss of continuity of the skin or body tissue that disrupts the integrity of the barrier function by rupturing a membrane and damaging the tissue beneath the skin [15]. The wound can be caused by physical, thermal, chemical, and radiogenic trauma. When a portion of the skin surface breaks, a wound is created, and wound healing begins to regenerate the integrity of the tissue and restore barrier function. The regenerating cells are guided towards a connective tissue scaffold to form normal, functional tissue structures. Various cell types and signaling molecules are involved in the repair and regeneration of the damaged tissue. For example, stem cells in the surrounding tissue can migrate to the site of the wound and differentiate into various cell types, such as fibroblasts, which produce collagen and other extracellular matrix proteins that contribute to the formation of a scaffold for tissue repair [16].

The classification of wounds is not a universal standard. Wounds are typically classified into categories depending on the cause of the wound, the duration of wound healing, and the depth of the wound [17,18]. Acute and chronic wounds are traditional terms for healing and non-healing wounds, classified according to the duration of wound healing. An acute wound has a normal healing process that is short and has no complications. The acute wound heals with primary intention and without tissue loss. Examples of acute wound healing include superficial traumatic wounds, first-degree burns, and surgical wounds [19]. When acute wound healing is interrupted and the proliferation process continues for more than four to six weeks, a chronic wound develops. The chronic wound heals with secondary intention, resulting in a lengthy healing process and complications due to some tissue loss that can affect many biological pathways [20]. Examples of chronic wound healing include pressure ulcers, diabetic foot ulcers, and venous ulcers.

### 2.1. Physiology of Wound Healing

Wound healing is a nonlinear process that proceeds forwards and backwards depending on various intrinsic and extrinsic factors [21]. In wound healing, tissue integrity is restored by replacing dead cells, the damaged extracellular matrix (ECM), and missing tissue structures with new cells and tissues, although the original function of the tissue is lost due to the complicated reconstruction process. Wound healing progresses by reducing the wound area and depth as new tissue forms to close the wound. Table 1 describes the wound healing process which can generally be divided into four phases: hemostasis, inflammation, proliferation, and remodeling.

### 2.2. Wound Tissue Types

**Figure 1:**
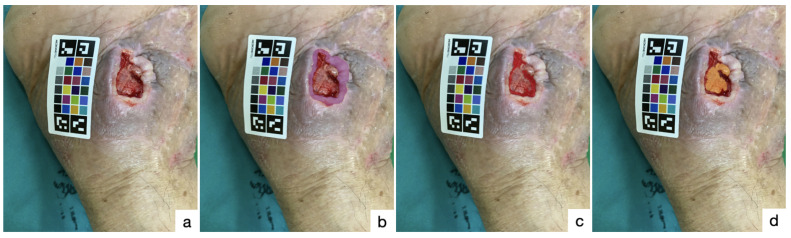
Wound tissue types: (**a**) wound without tissue labeling; (**b**) wound with epithelial tissue labeling in pink; (**c**) wound with granulation tissue labeling in red; and (**d**) wound with necrotic tissue labeling in orange. Our calibration chart was placed near the wound for color and measurement calibration.

The wound bed has various colors, including black, yellow, or red, with the color depending almost entirely on the type of tissue and the stage of wound healing. Granulation tissue, necrotic tissue, and epithelial tissue are the main tissue types that occur during wound healing. Table 2 summarizes the different wound tissue types. Figure 1 illustrates each wound tissue type on a sample image from our dataset.

### 2.3. Wound Assessment with Artificial Intelligence

The effective management of chronic wounds involves personalizing information about the underlying etiology and implementing appropriate wound management strategy. Reduction in the wound area is an important indicator for predicting wound healing. Seehan et al. [28] reported the correlation between the decrease in wound healing rate in the first four weeks and the complete healing after twelve weeks in diabetic foot ulcers and neuroischemic ulcers. The manual method of measuring the wound area requires wound care specialists to mark off the wound edge. This process is time-consuming, often inaccurate, prone to infection, and varies from one wound care specialist to another [8]. To overcome these limitations, algorithms based on convolutional neural networks (CNNs) have recently been used for automatic wound segmentation. It allows for higher accuracy, shorter timing, and a standardized protocol.

Several CNN architectures were introduced for image analysis tasks, including MobileNet [29], U-Net [30], ResNet [31], and EfficientNet [32]. Liu et al. [33] developed WoundSeg for wound segmentation based on MobileNet and VGG-16. The authors trained the model using their own dataset and the public Medetec Database. Their wound and non-wound labels were provided by physicians via their proprietary semi-automated tool developed using the Watershed algorithm. Their algorithm had a mean intersection over union (IoU) score of 84.60% and a Dice score of 91.66%. Wang et al. [5] proposed a wound area segmentation based on MobileNetV2, which requires low computational resources and a small number of parameters. They applied the model together with the connected component labeling (CCL), which enables the better detection of small connected components, thus increasing accuracy. The algorithms had a Dice score of 90.47% for their proprietary wound dataset of 1109 digital foot ulcer images and a Dice score of 94.05%. These aforementioned studies only focused on non-wound and wound labels on the public Medetec Wound Database.

The composition of the different tissue types in the wound could serve as an indicator of the wound healing progression. Quantitative wound assessment is critical for the management and monitoring of wound healing. Several studies have recently investigated the use of AI to characterize different wound tissue compositions. Pholberdee et al. [34] developed a deep learning algorithm for wound tissue segmentation that had IoU scores of 72%, 40%, and 53% for granulation, necrosis, and slough tissues, respectively. Ramachandram et al. [9] developed the AutoTrace and AutoTissue models using their proprietary Swift Medical Wound Dataset with 465,187 and 17,000 image-label pairs for wound area segmentation and wound tissue segmentation, respectively, with a test set of 58 chronic wound images. The model had IoU scores of 86.44% for segmenting the wound area and 71.92% for segmenting four different tissue types. The authors, however, did not report IoU scores for each tissue type. Not many studies have focused on wound tissue segmentation because there were no publicly available datasets of chronic wounds with such labels.

However, with the two-dimensional method of wound segmentation, it is sometimes difficult to capture entire wounds because of the curvatures of the human body. Several studies have focused on three-dimensional wound area measurement to improve the accuracy of the measurement and overcome this problem. Wannous et al. [35] proposed a workflow for assessing wounds using a combination of multiview tissue classification and 3D wound reconstruction. The first step involves using a multiview tissue classification algorithm to generate a segmented wound area from images taken from different views. The second step involves using a 3D wound reconstruction algorithm to generate a 3D model of the wound from the same set of images, using the resulting wound areas identified in the first step. Liu et al. [36] developed a method for the three-dimensional measurement of a wound area that involves structure from motion, least squares conformal mapping, and image segmentation. To create a single three-dimensional wound model, the method requires more than twenty wound images taken at difference angles of less than thirty degrees. The method has been shown to be more accurate than the 2D method for measuring wounds. Similarly, Barbosa et al. [37] presented a three-dimensional wound reconstruction method that involves image segmentation, structure from motion, and mesh reconstruction. The average error in measuring the wound area decreased as the number of images used to construct the three-dimensional wound model increased. The average error was 12.47% and 3.8% when two and ten images were used to create the three-dimensional model, respectively.

Although significant progress has been made in the development of algorithms for wound assessment, there are still areas for improvement. One of the biggest challenges is the ability of the algorithms to accurately segment. There are several approaches to wound segmentation that are commonly used, and the most appropriate method may depend on the specific context and requirements. One trend in wound segmentation is the use of CNNs, which have proven to be effective at quickly and accurately processing image data and classifying different wound tissues, such as the epithelialization area, granulation tissue, and necrotic tissue. The next challenge is the use of wound images acquired in an uncontrolled environment, meaning the images may not have been acquired under controlled lighting and positioning conditions. The accurate assessment of the wound depth from images is also challenging, as this information is important in determining the appropriate treatment. In addition, greater inter- and intra-physician agreement is needed when evaluating the performance of these algorithms. Finally, the robustness of the algorithms in longitudinal studies, where they are used to track the progression of a wound over time, is also an important consideration. Overcoming these challenges will be critical to the development of reliable and effective automatic wound assessment tools.

## 3. Materials and Methods

### 3.1. Clinical Study

This was a prospective study that included 31 wound images taken at different time points from 20 patients attending the wound clinic of Songklanagarind Hospital. All wound images were taken with a smartphone. We included adult patients over 18 years of age who had acute and chronic wounds, regardless of the duration and size of the wound. We excluded patients with circumferential wounds, scar wounds, and wounds with primary intention sutures. We also excluded end-of-life care patients who were unable to make their own decisions. All wound images were taken with the written informed consent of the participant before a photograph was taken.

Table 3 shows the patient characteristics in our study, including age, sex, wound type, wound site, exudate, wound amount, and dressing material. Our dataset contains mostly elderly people with a variety of wounds in our dataset.

### 3.2. Data Collection

When an image is taken with a smartphone, the exposure, white balance, ISO noise, and other camera settings are automatically adjusted to match the image itself and the environment around it. Different smartphone brands and models use different algorithms. Our goal is to develop an algorithm that can be applied to images taken with any smartphone camera. Since recent smartphones are capable of producing high-quality and high-resolution images, we did not impose any restrictions on the smartphone devices used to capture the images. To ensure that the results of our study were not affected by the type of device used to take the images, we used a color and measurement calibration chart, we employed color calibration techniques to normalizing images. We expected that these techniques may reduce the differences between images taken with different smartphones under different environmental and lighting conditions and thus allow for more accurate image analysis, especially for a small dataset.

**Figure 2:**
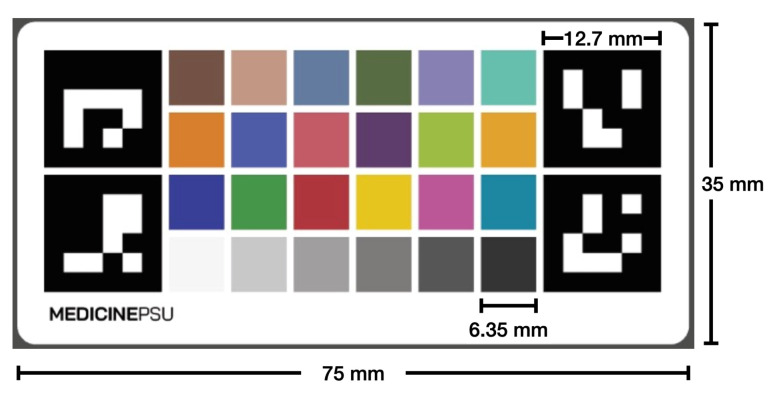
The calibration chart consists of four ArUco markers for the automatic detection and the Macbeth chart of 24 square color patches for color calibration.

We developed a calibration chart that has a Macbeth chart [38] and four ArUco markers at four corners (see Figure 2). The entire calibration chart has a dimension of 75 mm in width and 35 mm in length with four ArUco markers at each corner, whose dimension is 12.7 mm in both width and length, and 24 colored squares, whose dimension is 6.35 mm in both width and length for each square, arranged in a 6 × 4 grid that forms the Macbeth chart. The Macbeth chart consists of 24 square color patches with different spectral reflections that mimic those of natural objects. The Macbeth chart can help calibrate an image to have a consistent color appearance under different lighting conditions. It also helps in measuring the wound. Our color chart incorporates the ArUco markers, which help with the detection and alignment of the chart, allowing for automatic calibration to be performed.

**Figure 3:**
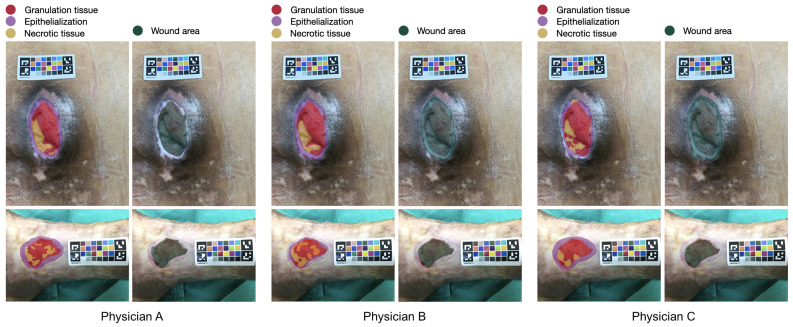
Example of a wound image labeled by three different physicians with the calibration chart positioned near the wound for color and measurement calibration. The red, orange, purple, yellow, and blue colors represent granulation tissue, necrotic tissue, epithelialization area, and wound area, respectively. Despite the use of clear guidelines and standards for labeling, slight differences in the areas annotated by the three physicians were observed. These differences may be due to a range of factors, including the physicians’ training, experience, and personal approaches to image interpretation.

All calibration charts were printed in the same batch using an industrial color laser printer on paper made from bleached eucalyptus kraft pulps. Color calibration was performed on the printer prior to printing. All calibration charts were autoclaved and sterilized before being used on the patient. The calibration chart was placed next to the wound before the image was taken. The photograph was taken without flash at a distance of approximately 30 centimeters from the wound. The image must contain the whole wound area and the entire calibration chart. All photographs were taken by physicians. There was no restriction on the brand or operating system of the smartphone, but it had to be able to capture an image with a resolution greater than 8 megapixels. Figure 3 shows examples of a wound image labeled by three different physicians. Despite clear guidelines for labeling, slight differences were noted in the areas labeled by the three physicians.

### 3.3. Data Annotation

The training of supervised the deep learning models to segment different wound tissue regions requires ground truth. In our study, physicians needs to annotate four different labels in each image: namely the epithelialization area, granulation tissue, necrotic tissue, and wound area. The first three labels (epithelialization area, granulation tissue, and necrotic tissue) are mutually exclusive. All physicians have more than ten years of practice experience. These labels were used for the development of deep learning models. The definitions for each label were agreed prior to data labeling by all physicians. The annotation was performed using the Supervisely web-based annotation tool (https://supervise.ly/ (accessed on 14 January 2023)) on a touchscreen tablet that allows the physician to draw fine lines around the designated label. After labeling, all physicians were met again and voted for the labels from one out of three physicians for each image under a blind test.

### 3.4. Wound Assessment Framework

**Figure 4:**
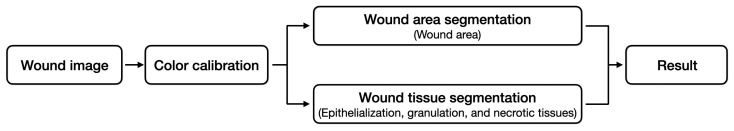
Wound assessment framework.

Our wound assessment framework (see Figure 4) consists of automatic color calibration, which normalizes the color of a wound image for more accurate image analysis; and automatic wound segmentation, which uses a deep learning model to determine the wound area, epithelialization area, granulation tissue, and necrotic tissue. Since the types of wound tissue are mutually exclusive and the wound area is the extent that covers the body surface where the skin is exposed to injury, we used a multi-task deep learning model that has two output branches: one for the wound area segmentation and the other for wound tissue segmentation. The results of the model can be converted to the real-world metric scale using the color and measurement calibration chart and can be used later to track the progress of the treatment.

### 3.5. Automatic Color Calibration

Details on the image taken with different smartphone cameras may vary due to camera configurations, environmental settings, lighting conditions, and the angle from which the photo was taken. These general issues may reduce the segmentation and measurement accuracy. Our calibration chart is specifically designed to solve the problem of color and scale discrepancies in the image. Our automatic color calibration is a two-step process consisting of the detection of ArUco markers and the correction of colors based on the Macbeth chart. The calibration chart also serves as a ruler for wound measurements. The process of color calibration can be performed automatically without human supervision or intervention.

#### 3.5.1. Detection of ArUco Markers

Our calibration chart contains four ArUco markers at each of the four corners and the 24-color Macbeth chart at the center. The ArUco markers are synthetic binary square fiducial markers with a black border and an inner identifiable binary matrix. The markers provide enough correspondence to determine the camera pose. The binary encoding makes them robust, with built-in error detection and correction capabilities. The ArUco markers are widely used for robot navigation and augmented reality, which benefits the development of the algorithms and makes them open source, real-time, and robust. We employed the ArUco library developed by Muñoz and Garrido [39] for the detection of square ArUco markers.

**Figure 5:**
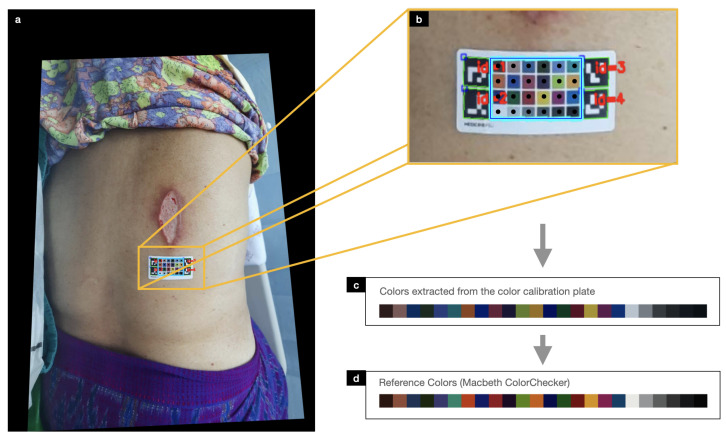
Color calibration process consists of (**a**,**b**) detecting the color and measurement chart using the ArUcO markers, (**c**) extracting all 24 color plates in the color and measurement chart, and (**d**) calibrating them against their reference colors.

ArUco marker detection consists of two steps: the detection of square shapes as marker candidates by adaptive thresholding, contour segmentation, and image filtering; and the decoding of the binary fiducial marker within the square. Once the four ArUco markers were detected, the image was first rotated in the same orientation based on the arrangement of the unique identification number in each ArUco marker. Camera position estimation was then performed to determine the camera position with respect to the ArUco marker. This resulted in rotation and translation vectors that were used to align the coordinate system of the camera with the coordinate system of the marker (see Figure 5a).

Since the location of the ArUco markers corresponds to the coordinates in the four corners of the MacBeth chart, a 6×4 grid table was first placed between the four makers to determine the components of the 24-color MacBeth chart (see Figure 5b). The mean color in the middle of each square grid was then obtained for calibration against its reference color in the next step.

#### 3.5.2. Color Correction

When wound images were taken under different lighting conditions, the color perception of the wound image may differ from a realistic wound. For example, when a wound image was captured in white light compared to warm light, the two images could have a different color range, with the image appearing yellowish in warm light. The calibration chart was designed to calibrate the color under the same conditions as a reference color.

**Figure 6:**
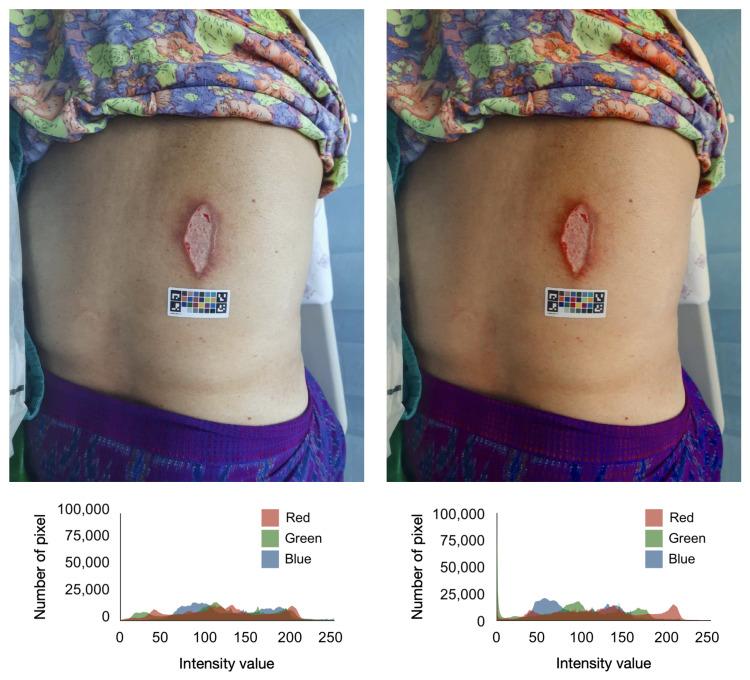
Comparison of the same wound image before and after color calibration.

Given a source matrix with 24 source RGB values extracted from a wound image using the method described in an earlier section and a target matrix with 24 reference RGB profiles, a Moore–Penrose inverse matrix was first computed to find the best possible solution for mapping the source RGB values to the target RGB values. Then, a color transformation matrix was calculated using the Moore–Penrose inverse matrix and the target matrix. The resulting color transformation matrix was then linearly applied to the source RGB values, resulting in the color-corrected image. After color calibration, the source and reference RGB values should be extremely linear. We employed PlantCV [40] which supports the analysis of color images for scientific research for color calibration. Figure 6 shows the comparison of the same wound image before and after color calibration.

### 3.6. Deep Learning for Wound Segmentation

**Figure 7:**
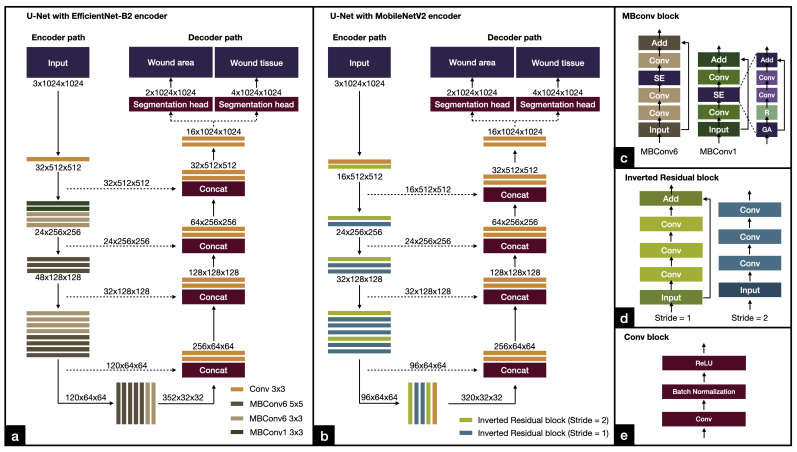
Architectures of our deep learning models. (**a**) U-Net with EfficientNet-B2. (**b**) U-Net with MobileNetV2. (**c**) MBConv block used by the EfficientNet-B2 encoder. (**d**) Inverted residual block used by the MobileNetV2 encoder. (**e**) Conv block.

We employed U-Net [30], a popular network architecture for semantic segmentation tasks that can work well with a small dataset, especially in the biomedical domain. U-Net is a U-shaped network consisting of an encoder and a decoder with skip connections between layers at the same level (see Figure 7). For each level, the encoder downsamples the feature maps by halving their spatial dimension and doubling the number of filter channels. The decoder upsamples the feature maps from the lower level and from the encoder at the same level by using a convolutional transpose layer with half the number of filter channels. Our model has two output branches, each receiving feature maps from the decoder to create segmentation masks. The first output branch is for segmenting the wound area (see Figure 7). The second output branch is for segmenting different types of wound tissue (epithelialization area, granulation tissue, and necrotic tissue). The number of outputs for the wound area segmentation branch was set to 2 (wound area and background) and for the wound tissue segmentation branch was set to 4 (three wound tissue types and background).

We followed the original implementation of U-Net [30]. Figure 7 shows the details of the architecture of our deep learning networks. We investigated two different encoders: EfficientNet and MobileNetV2. Both networks are computationally efficient. EfficientNet is a model that achieves good balance between accuracy and efficiency, while MobileNetV2 is a lightweight model specifically designed for efficient execution on small devices such as smartphones. By examining both EfficientNet and MobileNetV2, we can compare the performance of the algorithms and the suitability of the model for different applications.

#### 3.6.1. EfficientNet

EfficientNet [32] is a powerful and lightweight convolutional neural network architecture for computer vision that uses a compound coefficient to jointly scale up all the dimensions of the backbone network, rather than just independently adjusting a filter size or network depth. This results in a smaller number of parameters and thus faster training and inference time. EfficientNet uses the Mobile Inverted Residual Bottleneck Convolution (MBConv) building block, which consists of a combination of depthwise convolution, pointwise convolution, and expansion layers. The MBConv block first expands the input feature maps with a 1 × 1 convolution, then goes through a 3 × 3 depthwise convolution, and squeezes the feature maps with a 1 × 1 convolution (see Figure 7c). The MBConv block helps reduce the number of parameters and computational cost while maintaining the ability to learn complex features. The backbone network starts with a convolution, followed by multiple MBConv blocks that are sequentially arranged in seven stages, and ends with a convolution (see Figure 7a). The use of MBConv blocks in EfficientNet allows the model to be more efficient and accurate compared to other CNN models. Our work used EfficientNet-B2 which has 9.2 million parameters and 1.0 billion FLOPS. The model fits well with the computational resources we had available for training.

#### 3.6.2. MobileNetV2

MobileNetV2 [41] was designed for use on small devices such as smartphones. It employed MBConv as its standard building blocks. The backbone network starts with a convolution, followed by MBConv blocks arranged in seven stages, and ends with a convolution (see Figure 7b). Compared to EfficientNet, MobileNetV2 used MBConv on a smaller scale. MobileNetV2 has 2.1 million parameters with 0.3 billion FLOPS. The model is best suited for use on mobile devices.

#### 3.6.3. Network Training

In this study, we investigated two network variants: U-Net with EfficientNet and U-Net with MobileNetV2. For each network variant, we trained the dataset with and without color calibration applied to compare the effects of color calibration on the images.

Prior to training, wound images and associated ground truth labels were resized to 1280 by 1280 pixels by central cropping while maintaining an aspect ratio. We used data augmentation while training at random with a central cropping of 1024 by 1024 pixels, horizontal flipping, vertical flipping, grid distortion, brightness variation, contrast variation, and Gaussian noise addition. We used the albumentations library for data augmentation with default parameters.

All networks were trained using the Adam optimizer with a learning rate of $10^{-3}$, a weight decay of $10^{-4}$, and a batch size of four images. The networks were trained for 100 epochs using a pixel-wise weighted cross entropy loss with the model that has the lowest loss being kept aside.

#### 3.6.4. Model Evaluation

**Figure 8:**
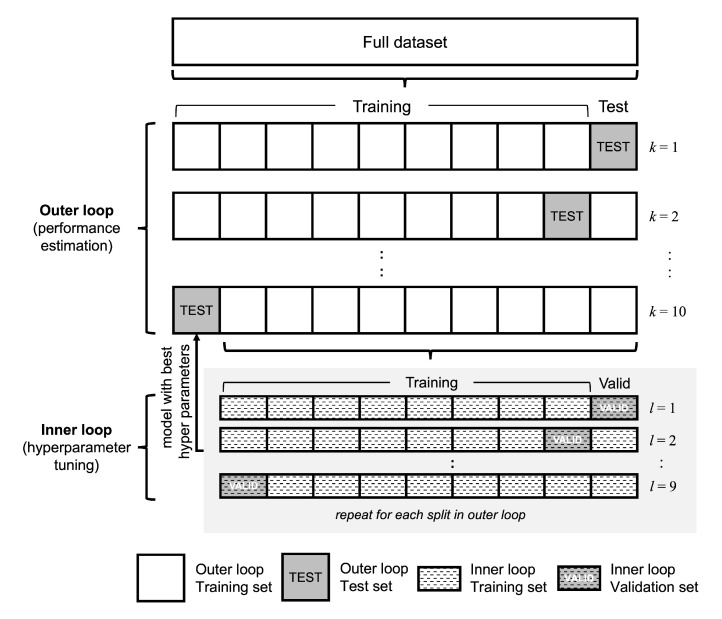
Diagram of nested cross validation with ten outer folds and nine inner folds that were used in this study. The nested cross validation scheme resulted in all images being used for testing.

We used nested cross-validation with 10 outer folds and 9 inner folds to train and evaluate the performance of the models in all experiments. Nested cross-validation performed multiple rounds of training and validation to optimize the model parameters and test the resulting model on an independent hold-out test set (see Figure 8). The dataset was divided into nested cross-validation folds at random, ensuring that each set had a roughly similar number of instances and contained unique subjects. We ensure that the images of the same patient are not distributed across different folds.

The nested cross-validation scheme allows us to develop and test our model on a limited sample size. It also has the benefits of better control of overfitting and providing a more reliable estimation. We used the same splits in the cross-validation folds for all experiments. Nested cross-validation is more appropriate when there is a limited number of images in the dataset. In addition, we can evaluate the performance of the approaches for all images in the dataset.

### 3.7. Evaluation Metrics

The two common metrics used to evaluate semantic segmentation algorithms are pixel accuracy and intersection over union (IoU). Assume that the number of correctly predicted wound pixels is called true positive (TP), the number of correctly predicted background pixels is called true negative (TN), the number of incorrectly predicted wound pixels is called false negative (FN), and the number of incorrectly predicted background pixels is called false positive (FP). Pixel accuracy is a measure used to compare the accuracy of image segmentation with ground truth. It is calculated using the ratio between the number of correctly predicted pixels and the total number of pixels:

(1) $$\mathrm{Pixel}\mathrm{Accuracy}=\frac{\mathrm{TP}+\mathrm{TN}}{\mathrm{TP}+\mathrm{FP}+\mathrm{TN}+\mathrm{FN}}.$$

Intersection over union (IoU) is a standard metric to evaluate an area over which the prediction and ground truth overlap. This method is calculating the number of pixel intersections divide by the total number of pixels between the prediction and ground truth:

(2) $$\mathrm{Intersection}\mathrm{over}\mathrm{Union}=\frac{\mathrm{TP}}{\mathrm{TP}+\mathrm{FP}+\mathrm{FN}}.$$

#### Environment Setup

All experiments were performed on a workstation with eight-core processor CPU, 64-GB RAM, and an Nvidia RTX A5000 24-GB GPU. We used Python v3.8.12, Pytorch v1.11.0, CUDA v11.3, OpenCV v4.5.4.58, PlantCV v.3.13.0, SegmentationModels v0.2.0, albumentations v1.0.3, and scikit-learn v1.0.1.

## 4. Results

### 4.1. Inter-Rater Agreement

Table 4 shows the comparison of inter-rater agreement between a pair of physicians on each class label using the IoU measure. The mean inter-rater agreement for the wound area was 0.6147, the epithelialization area was 0.2822, the granulation tissue was 0.5733, and the necrotic tissue was 0.2720. Agreement scores for each wound feature were similar with good agreement for the wound area and granulation tissue and moderate agreement for the epithelialization area and necrotic tissue.

### 4.2. Wound Area Segmentation

**Figure 9:**
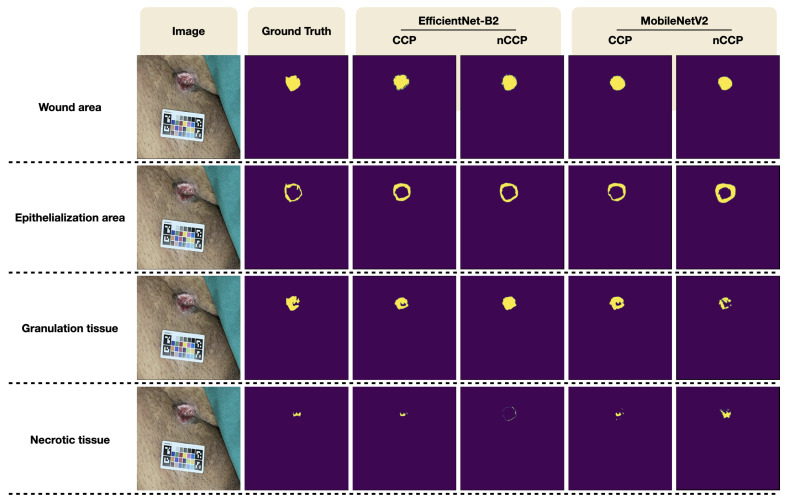
Qualitative segmentation results from our proposed method.

Table 5 shows the performance of our models in terms of IoU score and pixel accuracy. Scores were calculated from all images in the dataset using the nested cross-validation scheme. For the segmentation of wound area, U-Net with MobileNetV2 and U-Net with EfficientNet-B2 trained and evaluated with the original wound images without color calibration achieved a mean IoU of 0.6624 ± 0.2307 and 0.6878 ± 0.1359, respectively, and a mean pixel accuracy of 0.9854 ± 0.0219 and 0.9871 ± 0.0101, respectively. Performance improvement was observed when the network was trained on the wound images after color calibration was performed. With MacBeth color calibration, U-Net with MobileNetV2 and U-Net with EfficientNet-B2 achieved a mean IoU of 0.6826 ± 0.1987 and 0.6964 ± 0.1359, and a mean pixel accuracy of 0.9881 ± 0.0105 and 0.9866 ± 0.0114, respectively, (see Figure 9). In general, U-Net with EfficientNet-B2 had better performance than U-Net with MobileNetV2.

### 4.3. Wound Tissue Segmentation

A wound is usually composed of different tissue types which are related to the stage of wound healing. Our models were trained to segment the epithelialization area, granulation tissue, and necrotic tissue from the wound image. For the segmentation of different wound tissues, the highest IoU score and pixel accuracy were found in granulation tissue, followed by epithelialization area and the necrotic tissue. Color calibration has also benefited the segmentation of different wound tissues. The model trained on wound images with color calibration had a higher performance compared to the model without color calibration. U-Net with EfficientNet-B2 has a higher mean IoU and pixel accuracy than U-Net with MobileNetV2 for all experiments. U-Net with EfficientNet-B2 trained and evaluated on color-calibrated wound images had a mean IoU of 0.3957 ± 0.1869, 0.6421 ± 0.2444, 0.1552 ± 0.1428 for epithelialization area, granulation tissue, and necrotic tissue, respectively, and a mean pixel accuracy of 0.9808 ± 0.0157, 0.9920 ± 0.0064, and 0.9853 ± 0.0123 for the epithelialization area, granulation tissue, and necrotic tissue, respectively, (see Figure 9).

### 4.4. Longitudinal Wound Assessment

**Figure 10:**
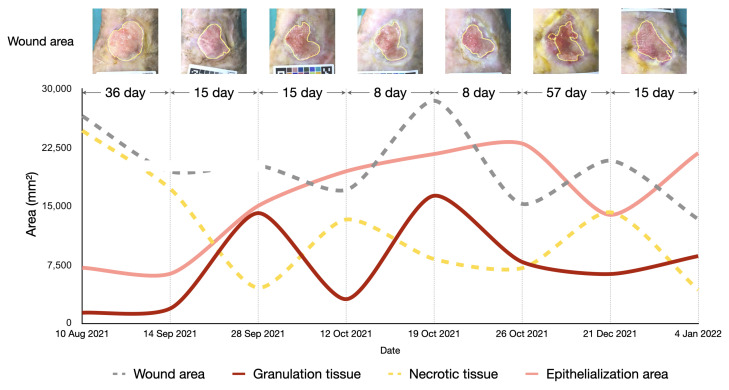
Assessment of wound healing over time using our algorithms with a patient who visited the wound clinic seven times. The area of the wound, epithelialization, granulation tissue, and necrotic tissue can be derived from the wound images. The resulting information can be used by physicians to improve wound care.

The area of the wound and the different tissue types is represented by the number of pixels, which can be converted into square millimeters using the reference size from the calibration chart. Figure 10 shows the results of longitudinal wound assessment of a patient who visited the wound clinic seven times. We applied our best performing model to segment the wound area and different tissue types from the wound image taken at each visit. We then converted the pixel area into square millimeters. The graphs show the changes in wound area and wound tissues over the course of the treatment. The area of wound and necrotic tissue tended to decrease slightly over time, whereas the area of the granulation tissue and epithelialization area tended to increase slightly over time. There was some variations in area because the patient had a chronic wound, which is typically difficult to heal. This information can be utilized by physicians to improve wound care.

## 5. Discussion

### 5.1. Wound Segmentation

This study aimed to develop an AI-assisted wound assessment tool to help physicians track the progress of wound healing and adjust treatment as needed. To this end, an automatic color and measurement calibration process and a multi-task deep learning model were developed. The calibration chart placed in the image can be automatically detected by our approach and all color chips can then be identified. The colors in the image can be calibrated against their reference colors and the measurement can be quantified with respect to the reference color grid. Our deep learning model can provide an estimate of wound area and wound tissue from a wound image taken with any smartphone.

The challenge of this study is that, to train our deep learning models, we need goldstandard wound labels provided by physicians; however, the physician assessment of wounds is particularly subjective [8]. Although we made our wound definitions clear before data annotation, we obtained moderate agreement scores among physicians for the annotation of wound area and different tissue types. We argued that wound tracings are time-consuming, prone to errors, and inherently difficult. In addition, similar results on inter-rater agreement were obtained by Howell et al. [8] when they compared wound tracings among physicians. We therefore decided to have another round of discussion with the physicians after all the wound images were annotated and the agreement between the physicians was calculated. We asked each physician to blindly vote for one of the three wound labels that they felt most closely matched the wound definitions and was most appropriate for training the algorithm. We think that this is the most appropriate way to deal with the problem when the agreement between the assessors was not consistent.

The wound area plays an important role in assessing wound healing. The standardized measurement of the wound area allows physicians to track the progression of wound healing. Our models were able to segment the wound area from a wound image with good accuracy. The performance of U-Net with MoblieNetV2 was only slightly lower than that of U-Net with EfficientNet-B2, but with a much smaller number of parameters, allowing the model to run on mobile devices. Our results were similar to those of Scebba et al. [42], Pholberdee et al. [34], and Ramachandram et al. [9].

Different types of wound tissue served as indicators for assessing the stage of wound healing, which precisely indicated the patient’s response to wound care. Our wound segmentation model can segment three main tissues that are present in the wound region: the epithelialization area, granulation tissue, and necrotic tissue. Our model performed well in detecting granulation tissue, which appears as a large region on the wound image and is present in all images in our dataset. Performance in the epithelialization area was moderate. This could also be due to the imbalance in classes, small dataset, and inconsistent data annotation across the wound images. In contrast, our model had a poor IoU score for necrotic tissue. We suspect that this is because the area of necrotic tissue in each image is small and only a small number of images contain necrotic tissue. The performance of our models was similar to those of Ramachandram et al. [9] and Pholberdee et al. [34], who also reported poor performance with the epithelialization area and necrotic tissue.

### 5.2. Effects of Color and Measurement Calibration

Lighting is an important factor to consider when taking photographs, as it directly affects the appearance of the colors in the image. The wound generally appears in many colors because it is made up of many types of wound tissue. The most common colors of the wound are red, black, yellow, pale pink, and white. These colors form the appearance of the wound. In addition, lighting conditions affect the hue of the wound, which could result in different wound images if the picture of wound is taken under different lighting conditions. This could affect the precision of wound segmentation algorithms [8,43]. To improve the accuracy of wound segmentation, we developed the calibration chart and captured all wound images with the calibration chart. Our color calibration algorithm ensures that all wound images have similar color shades. Wound segmentation models trained and evaluated with color-calibrated wound images had higher scores than those trained and evaluated with original wound images. Apart from color calibration, using the color grid in our calibration chart, we were able to correctly quantify the size and area of the wound in relation to the size of the color chip.

### 5.3. Longitudinal Wound Assessment

Our wound measurement approach can automatically segment the area of the wound, granulation tissue, epithelialization area, and necrotic tissue on a wound image and give measurements with millimeter accuracy. It provides several advantages compared to a traditional wound measurement that involves measuring the width and length of the wound with a ruler and calculating the wound area by multiplying the length and width. The traditional method appears to lead to overestimation, especially in an irregular surface of the wound area [44]. Chan et al. [45] reported that the automatic measurement of length, width, and wound area with artificial intelligence can improve the problem of overestimation in traditional wound measurement. In our method, the wound area was calculated from the wound prediction output of the models converted from the area in pixels to millimeters using the reference length from the calibration chart. This technique is suitable for regular wound surface and could represent a realistic wound area. The automated measurement of a wound area with artificial intelligence can help clinicians track the progress of wound healing from anywhere, reducing the time and effort and increasing the efficiency of wound care.

### 5.4. Comparison to Other Studies

Many recent studies have investigated approaches for assessing wounds. Several studies [5,33] conducted on the Medetec Wound Database of open wounds have only the wound area as a label. They did not consider the different types of wound tissue. Few studies investigated the segmentation of different wound tissue types [9,34]. Pholberdee et al. reported results similar to ours in that IoU scores for segmenting necrosis and slough tissue were lower than those for granulation tissue. It should be noted that Howell et al. [8] compared the tracing of wound tissue among physicians and found that agreement on wound characteristics can vary considerably among wound care specialists. This was also reflected in the inter-rater agreement among the physicians in our study. Recently, Ramachandram et al. [9] evaluated their proprietary algorithm but did not report how their calibration sticker worked or how their algorithm performed in the long run. We highlighted that our inter-rater agreement for different wound tissue types was in the same trend as [8,9]. Despite the fact that there are commercial wound assessment applications on the market, the results of our study, together with other studies, highlight the difficulties arising from the different criteria for wound assessment, which vary from clinician to clinician and make it difficult to establish a consensus. Those wishing to use a commercial application may need to consider the white paper published by the company, which may reflect the performance and characteristics of the algorithm.

### 5.5. Limitations

The main limitation of our study are the small dataset and the imbalance classes [46]. First, the small wound dataset would directly affect the performance of the wound segmentation model. With more cases, the model could learn from more examples, which would lead to more accurate results. We addressed this issue by implementing the nested cross-validation scheme as our evaluation method so that all images were used for training, validation, and testing. The results reported in this study were obtained from all cases. We also used extensive data augmentation during training to reduce the impact of the small dataset. We suspected that these low scores in some tissue types are due to the small sample size we have, so the model did not generalize well to general images. Then, our wound segmentation dataset contains imbalanced class labels that could affect algorithm performance. Our dataset contains the labels of the epithelialization area and granulation tissue in almost all images, but only a few necrotic tissue labels. In addition, the epithelialization area and granulation tissue in the images appear very large compared with the very small necrotic tissue. Although we assigned class weights to the loss function during training, this problem still severely affects the performance of the algorithm. Our model performs better in the segmenting granulation tissue and epithelialization area than the necrotic tissue. In addition, additional information about the tissue being imaged can be captured through infrared imaging, which can be used to identify the areas of inflammation that may be indicative of a wound or other abnormality. The use of thermo-sensitive films can also be used to detect changes in temperature and moisture levels. These films can be used to identify the areas of increased blood flow, which may indicate the presence of a wound or other abnormality through computational modeling [47]. Finally, since physicians are involved in the decision-making process regarding wound assessment, it is good to compare the results of the algorithms with the physicians’ assessments. This could include both inter-physician and intra-physician assessment, comparing the results of the algorithms to those of multiple physicians or to those of a single physician at different points in time. This would help ensure that the results of the algorithms are consistent with the assessment by physicians. As such, the algorithm can be trusted to make reliable decisions.

### 5.6. Future Work

In the future, we plan to further improve our approach by collecting additional image data to further fine-tune the deep learning models. This could be achieved by making research data collection a routine task in wound clinics. We also plan to address the challenge of estimating the depth of the wound, which is an important factor in evaluating the healing process. We consider the use of depth cameras to capture more information about the 3D structure of the wound. In addition, we plan to develop a handheld device that incorporates our AI algorithms for wound assessment, allowing physicians to easily and accurately assess wounds in different environments. This device could be particularly useful in remote areas where access to specialized wound care facilities is limited. Our goal is to develop a reliable, user-friendly device that can assist physicians in providing optimal wound care to their patients.

## 6. Conclusions

Proper wound management requires routine wound measurement and assessment that need to be accurate, precise, and repeatable. In this study, we developed an approach for automated wound assessment that involves placing a calibration chart near the wound, taking an image of the wound with a smartphone, and analyzing the image using AI. To start with, wound images were collected from patients visiting the wound clinic at Songklanagarind Hospital. The wound area and three important wound tissues on the images were annotated by three physicians with ten years of experience. A deep learning model was then developed to mimic what the physicians did on the images. Our deep-learning model was able to accurately the segment wound area and granulation tissue, but was inconsistent with respect to the epithelialization area and necrotic tissue. Although we used nested cross-validation, which helps in developing an algorithm on a small dataset, more images are needed for the algorithm to learn to better segment small and variable tissue areas such as necrotic tissue. The calibration chart, which helps calibrate colors and scales, can help improve the algorithm performance. The algorithm could allow the longitudinal assessment of the wound, which could help clinicians tailor treatment to the patient’s condition. The resulting approach can be developed into an application or a tool to help physicians assess wound healing accurately, easily, and anywhere.

## Figures and Tables

**Table 1:** Four phases of wound healing.

Phase	Description
**Hemostasis**	During wound development, the loss of tissue integrity results in bleeding that exposes the blood to various extracellular matrix (ECM) components. The bleeding is stopped by vasoconstriction and platelet plugging, which causes blood vessels to constrict, blood flow to slow or block, and platelets to aggregate. This leads to hemostasis and fibrin formation. Fibrin acts as a temporary matrix for cell migration. Fibrinolysis is then activated to dissolve the fibrin and provide space for further cell migration into the next phase of wound healing [22].
**Inflammation**	At the onset of inflammation, fibrin is broken down and vessels become permeable, releasing plasma and allowing inflammatory cells (neutrophils and macrophages) to migrate to the wound site. Neutrophils arrive within 24–48 h and help defend against infection by phagocytizing debris and pathogens. Macrophages, derived from blood monocytes, arrive 24–36 h later and aid in the removal of bacteria and debris by phagocytosis. They also secrete growth factors and cytokines that stimulate new blood vessel formation, granulation, and re-epithelialization [23].
**Proliferation**	The proliferation phase mainly involves the formation of granulation tissue and the immigration of keratinocytes on the wound bed. Keratinocytes stimulate the regeneration of epithelial tissue and restore the continuity of the epidermal layer. New tissue forms from a matrix of collagen, elastin, glycosaminoglycans, and other fibrous proteins, as well as fibrin and fibronectin. Fibroblasts produce and deposit extracellular proteins, including growth factors and angiogenic factors, which control the cell proliferation and angiogenesis [23].
**Remodeling**	The remodeling phase begins when collagen synthesis and degradation are balanced and the wound has closed. During this phase, the body replaces the temporary tissue formed during the inflammation and proliferation phases with new, stronger tissue. It also works to improve the appearance of the scar and adjust the tension of the scar tissue to match the surrounding tissue [24].

**Table 2:** Three types of wound tissue.

Tissue Type	Description
**Granulation tissue**	New connective tissue and microvessels form the granulation tissue, which is the main carrier of oxygen and nutrients during the healing process. The characteristic of granulation tissue is a rough and moist surface on the wound bed. Granulation tissue appears pale pink in the wound bed or bright, beefy red as the depth of the wound bed increases. In addition, granulation tissue provides a scaffold for the epithelialization process to form and cover the wound.
**Epithelial tissue**	Epithelialization is a complex process involving morphogenesis, cell proliferation, cell differentiation, and migration with substantial mitotic activity at the wound edge [25]. New epithelial tissue appears deep pink at the wound site as basal keratinocytes migrate to the wound surface or the epidermis regenerates. New epithelial tissue covers the wound from the edge to the center. Postoperative wound dressings have been used to accelerate and optimize the healing process by providing protection from contaminants, absorbing exudate and humidity, and promoting autolytic debridement [26].
**Necrotic tissue**	Necrosis is the death of body tissue. Cell death occurs due to lack of oxygen and interrupted blood supply for a long enough time [27]. Necrotic tissue can be divided into two main types: eschar is a dry, dark tissue that can thickly cover a wound bed; and slough is a soft, moist tissue that contains dead tissue and bacteria. Its color may range from yellow to green to tan to brown. Necrosis tissue cannot be salvaged and must be removed by appropriate wound debridement techniques to allow wound healing to occur [16,26].

**Table 3:** Patient characteristics.

Characteristic	Value
Age, mean ± SD [Range]	64.61 ± 20.41
	[17–85]
Sex, N (%)	
Male	11 (55.00)
Female	9 (45.00)
Wound type, N (%)	
Infection/inflammation	14 (45.16)
Pressure ulcer	8 (25.81)
Burn	4 (12.90)
Trauma	4 (12.90)
Diabetics	1 (3.23)
Wound location, N (%)	
Lower extremity	17 (54.84)
Foot (excluding heel)	5 (16.13)
Sacrum/coccyx	5 (16.13)
Upper/lower back	3 (9.68)
Chest/breast	1 (3.23)
Exudate, N (%)	
Serous	7 (22.58)
Purulent	1 (3.23)
Serosanguinous	1 (3.23)
Amount, N (%)	
Minimal	14 (45.16)
Moderate	3 (9.68)
Dressing material, N (%)	
Foam	8 (25.81)
Gel	7 (22.58)
Non-adhesive mesh	6 (19.35)
Aquacel Ag	3 (9.68)
Foam/non-adhesive mesh	1 (3.23)
Gauze	1 (3.23)
Granudacyn solution	1 (3.23)

**Table 4:** Inter-rater agreement between physicians on each class label.

Feature	Physicians	Intersection over Union	
		Mean	S.D.
Wound area	A vs. B	0.6739	0.2374
	A vs. C	0.5143	0.2303
	B vs. C	0.6559	0.2483
Epithelialization area	A vs. B	0.3248	0.2087
	A vs. C	0.2850	0.2609
	B vs. C	0.2368	0.2261
Granulation tissue	A vs. B	0.5438	0.3316
	A vs. C	0.5561	0.2303
	B vs. C	0.6201	0.2877
Necrotic tissue	A vs. B	0.2680	0.2763
	A vs. C	0.1635	0.2473
	B vs. C	0.1125	0.1991

**Table 5:** Performance deep learning models for wound area and wound tissue segmentation.

Feature	Architecture	Encoder	Color Calibration	Intersection over Union		Pixel Accuracy	
				Mean	S.D.	Mean	S.D.
Wound area	U-Net	MobileNetV2	Yes	0.6826	0.1987	0.9881	0.0105
			No	0.6624	0.2307	0.9854	0.0219
		EfficientNet-B2	Yes	0.6964	0.1359	0.9866	0.0114
			No	0.6878	0.1665	0.9871	0.0101
Epithelialization area	U-Net	MobileNetV2	Yes	0.3186	0.1630	0.9792	0.0153
			No	0.2854	0.1552	0.9770	0.0179
		EfficientNet-B2	Yes	0.3957	0.1869	0.9808	0.0157
			No	0.3672	0.1517	0.9812	0.0145
Granulation tissue	U-Net	MobileNetV2	Yes	0.5997	0.2885	0.9899	0.0086
			No	0.5879	0.2633	0.9921	0.0048
		EfficientNet-B2	Yes	0.6421	0.2444	0.9920	0.0064
			No	0.6297	0.2529	0.9915	0.0084
Necrotic tissue	U-Net	MobileNetV2	Yes	0.1386	0.1467	0.9831	0.0133
			No	0.1185	0.1754	0.9858	0.0125
		EfficientNet-B2	Yes	0.1552	0.1428	0.9853	0.0123
			No	0.1195	0.1040	0.9896	0.0073

## Data Availability

The data presented in this study are available upon request from the corresponding author. The data are not publicly available due to the institutional policy.
